# Exploring carer burden amongst those caring for a child or adolescent with an eating disorder during COVID-19

**DOI:** 10.1186/s40337-021-00485-7

**Published:** 2021-10-03

**Authors:** Kristen Maunder, Fiona McNicholas

**Affiliations:** 1 School of Medicine and Medical Science, University College Dublin, Dublin 4, Belfield, Republic of Ireland; 2 Department of Child and Adolescent Psychiatry, Our Lady’s Children’s Hospital Crumlin, Dublin 12, Crumlin, Republic of Ireland; 3 Lucena Clinic Services, Rathgar, Rathfarnham, Dublin 6, Republic of Ireland

**Keywords:** Eating disorders, Children and adolescents, Carers, Carer burden, COVID-19

## Abstract

**Background:**

Carer burden amongst carers of youth with an eating disorder is substantial and if not addressed can lead to negative outcomes for the patient, carer and family. The Coronavirus Disease 2019 (COVID-19) pandemic has made caring for youth with an ED even more onerous and preliminary research is beginning to emerge demonstrating the profound negative impact the pandemic is having upon individuals with EDs and their carers.

**Main:**

In this review, we briefly summarize what is known about carer burden in families where a young person has an ED, consider the additional impact consequent to COVID-19 and highlight the need for interventions aimed at alleviating this. Pre-COVID-19 research identifies high levels of psychological and physical strain amongst those caring for a child with an ED. Themes are beginning to emerge as to why COVID-19 may further exacerbate carer burden: (1) reduced access to ED services; (2) increased physical vulnerability and exacerbation of psychiatric co-morbidity amongst youth with EDs; (3) increased practical demands placed on carers; and (4) social isolation and decreased social support.

**Conclusion:**

The COVID-19 pandemic poses a specific threat to the mental health of youth with EDs and their carers. Given the salient role families play in caring for youth with an ED, attending to carer burden is imperative. Supporting carers through all phases of their child’s ED journey by offering adaptive and flexible supportive services which accommodate time constraints, geographic barriers and possible COVID-19 spread is essential.

## Plain English summary

Caring for a child with an Eating Disorder (ED) can be difficult at the best of times. Research is beginning to emerge demonstrating the negative impact the Coronavirus Disease 2019 (COVID-19) pandemic is having both on youth with an ED and their carers. In this review, we briefly summarize what is known about carer burden in families where a young person has an ED, consider the additional impact of COVID-19 and highlight the need for further supportive interventions. This review indicates that carer burden is substantial amongst those caring for a loved one with an ED and additional strain has been placed on carers during the pandemic. Key themes have emerged likely contributing to an increase in carer burden during COVID-19: (1) reduced access to ED services; (2) increased physical vulnerability and exacerbation of psychiatric co-morbidity amongst youth with EDs; (3) increased practical demands placed on carers; and (4) social isolation and decreased social support. Findings from this review aim to inspire further research on how best we can support carers of youth with EDs during the pandemic and beyond.

## Background

Eating Disorders (ED) have the highest mortality rate of all mental illnesses and commonly develop during adolescence [1]. The prevalence of EDs is growing [1], while the age of onset is decreasing with EDs increasingly being recognized in children as young as 5 years [2]. The prognosis for those with an ED varies, with many children and adolescents going untreated, not recovering or reaching only partial recovery [3]. With a median duration of illness of 6–7 years [4], significant strain can be placed on parents and the family unit. The associated carer burden can lead to adverse personal, family and patient outcomes.

The Coronavirus Disease 2019 (COVID-19) pandemic has created unprecedented levels of stress for all. The high transmissibility, morbidity and mortality amongst vulnerable groups has driven extreme public health measures to help control the spread. Uncertainty surrounding the disease and the imposition of mass home-confinement directives (including quarantine and isolation), are recognized to have potential for immediate and longer term collateral psychosocial consequences surpassing the medical sequalae [5]. These restrictive measures disproportionately affect children and adolescents given school closures, confinement of family members and limited opportunities for social engagement.

After enduring the impacts of COVID-19 for over a year, data is emerging attesting to the adverse mental health effects of the pandemic [6]. Research suggests a profound negative impact upon both individuals with EDs [7] and their carers [8]. In Canada, The National Eating Disorder Information Center experienced an increased number of contacts from carers and increased self-reported eating psychopathology during the pandemic compared to previous years [9]. Amongst those identifying with an ED, rates of anxiety and depression were also significantly higher [9]. Rates of pediatric admissions for Anorexia Nervosa (AN) have also increased during the pandemic [10]. The authors hypothesized a combination of social isolation, school closures and fewer opportunities for positive and distracting extracurricular activities has disconnected patients with EDs from protective factors, hence making room for ED cognitions and co-morbid mood disorders to intensify.

A recent qualitative study carried out in the United Kingdom, examined the impact of COVID-19 on both adults with AN and their carers. During the pandemic, carers reported a sense of increased responsibility for the well-being of their loved ones, in part driven by difficulty accessing services and also managing their own and their family’s well-being [8]. With a large body of literature highlighting the importance of the family emotional climate in both emergence and relapse of EDs [11], COVID-19 associated family adversity poses a specific threat to the mental health of youth with EDs and that of their carers.

In this review, we briefly summarize what is known about carer burden in families where a young person has an ED. We then identify key themes that are beginning to emerge highlighting why COVID-19 may further exacerbate carer burden amongst those caring for a child with an ED. Lastly, we highlight the need for interventions aimed at alleviating carer burden and propose possible supports that could be offered to carers during the COVID-19 pandemic and beyond.

## Carer burden amongst carers of youth with eating disorders

Carer burden is an all-encompassing term used to describe the physical, emotional and financial toll associated with providing care [12]. Caring for a child with a mental illness can be particularly challenging given the unpredictable nature and chronicity associated with many mental illnesses [13]. Disruption of everyday life routine, stigma and blame, financial difficulties as well as physical and emotional strain contribute to the burden placed on carers of youth with mental illness [14]. The degree of carer burden varies with amount of time carers spend with their child and the nature and severity of their mental illness [15].

The first edition of the Diagnostic and Statistical Manual of Mental Disorders (DSM-5) recognizes four common categories of EDs in youth: Anorexia Nervosa (AN), Binge Eating Disorder (BED), Bulimia Nervosa (BN) and Avoidant Restrictive Food Intake Disorder (ARFID). These EDs are characterized by disturbed eating behavior and distorted views regarding body weight and shape, and in the case of ARFID a disinterest in food, phobic avoidance or avoidance due to sensory aspects of food [16]. All EDs can cause impairments in psychological, physical and social functioning and can evade diagnosis for months or even years.

Core ED psychopathology, which involves an inability or unwillingness to eat, leading to malnourishment and medical compromise, is so alien to normal parenting practice that it can immobilize and incapacitate parents at a very early stage. Carers may find themselves in situations where they lack the resources or skills to cope with the various caregiving demands and must balance their new role as a carer with previous and existing roles, such as addressing family needs and paid employment [17]. Carer burden amongst parents of youth with EDs is considerable and if not addressed can lead to negative outcomes for the patient, carer and family.

## Unique challenges faced by carers of youth with eating disorders during COVID-19

Pre-COVID-19, research identified high levels of physical and psychological strain amongst carers of patients with EDs [18], with some studies suggesting carers experience higher levels of anxiety, depression and perceived carer burden compared to carers of other mental illnesses [19, 20]. The COVID-19 period has made caring for a child with an ED even more onerous. Available data from previous pandemics, such as the Southeast Asian Respiratory Syndrome (SARS) and H1N1 Influenza (H1N1) reveal higher levels of anxiety and distress, often attributed to isolation in the general public [21]. Whilst the long-term mental health implications of COVID-19 remain unknown, themes are emerging as to why COVID-19 may further exacerbate carer burden amongst those caring for a child with an ED. These themes are outlined below.

### Reduced access to eating disorder services

Given the highly contagious and transmissible nature of COVID-19, access to face-to-face ED treatment has been greatly reduced during the pandemic. To contain the virus and protect vulnerable people, especially those medically compromised, outpatient and inpatient ED care has been kept to a minimum while waitlists for treatment have grown. As a result, carers have become more involved than previously in their child’s care and have had to take more responsibility in both refeeding and monitoring their child’s physical and mental health status [22].

During the peak stage of COVID-19, some psychiatric wards for children and adolescents were forced to close in order to provide care for COVID-19 patients [23]. Carers of patients with EDs have expressed fears over premature discharge from services and described a rushed transition process with inadequate treatment plans being in place [8]. The shift from face-to-face to online telehealth sessions, has required the assistance of carers who have been given the added responsibility of weighing their child, recording food intake to allow for daily caloric estimates to be calculated and collecting vital signs when possible [22]. Carers may feel out of their depth and overwhelmed by these increased demands further contributing to carer burden.

A study carried out in Madrid, Spain presented COVID-19 related adaptations to treatment protocols within their child and adolescent ED program during the initial eight-week confinement period [23]. They also examined changes in clinical and treatment variables within their outpatient, day hospital and inpatient settings. During this time, there was a swift move from face-to-face to telehealth within the outpatient and day hospital settings with limited number of children being seen face-to-face when clinically indicated. Compared to the previous year, fewer new patients were evaluated within the outpatient setting and a larger majority of patients admitted were more medically compromised and had a previous history of inpatient admission. Within the inpatient program, strict social distancing and infection control measures were implemented. Therapeutic outings with families were discontinued, parents could no longer participate in meals on the ward, psychotherapy groups were cancelled and permission to visit the home during the last stage of hospitalization was discontinued. With respect to hospitalizations, 68% of patients and their families identified the onset of confinement as a possible precipitating factor for admission while 32% of adolescents reported an increase in family conflict during this time. Compared to the previous year, patients admitted to hospital presented with greater psychiatric co-morbidity (especially affective disorders) and suicide risk which the authors attributed to increased family conflict consequent to confinement within the home during the COVID-19 pandemic.

As a result of adaptations made in response to COVID-19, there has been a substantial curtailing of traditional evidence-based care offered and delivered to patients with EDs and their families. Having fewer face-to-face reviews, due to COVID-19 precautions, increases the risk of clinicians failing to pick up on early warning signs of clinical deterioration amongst patients and necessary treatment being delayed. This is of particular concern in youth with EDs, where medical stability is a recognized risk, and where disclosure of physical deterioration is often not volunteered by the patient, and easily camouflaged by the patient with baggy attire. More restricted access to inpatient, outpatient and day hospital programs might also mean vulnerable young people in the community with new onset of an ED (or yet undiagnosed) are unable to access treatment or exposed to long delays, whilst their mental and physical state deteriorates rapidly. Premature discharge from inpatient services, in response to necessary triaging and making room for new intake, could also increase relapse, a need for readmission and an undermining of carer confidence in home management. The long-term implications of reduced access to ED services and added responsibility placed on carers needs to be evaluated. This is occurring simultaneously to data suggesting an increase in prevalence of EDs [10].

### Youth with eating disorders deemed high medical risk

The clinical complexity of EDs further compounds carer burden, and this has been exacerbated during COVID-19. Patients with AN or BN are considered high medical risk given their propensity towards electrolyte abnormalities, cardiac compromise and decreased bone marrow function lowering immunity. Nutritional deficiencies are common, in particular deficiencies in Vitamin D, calcium and phosphate. Studies are beginning to emerge demonstrating a correlation between Vitamin D status and COVID-19. Vitamin D has been claimed as potentially protective against the infection given it may be associated with immune-competence and inflammation involved in influencing the outcomes of COVID-19 [24]. Poor Vitamin D status is associated with an increased risk of COVID-19 infection [24] while substantial evidence supports a link between Vitamin D deficiency and COVID-19 severity [25]. Two ecological studies have demonstrated an inverse correlation between national Vitamin D status and COVID-19 mortality in European countries [26, 27]. As sun exposure has been cited as one of the best sources of Vitamin D, individuals confined to their homes during COVID-19 limits sun exposure, further contributing to Vitamin D deficiency. Vitamin D deficiency has been linked to severity of childhood respiratory illness [25], highlighting the need to consider if an association exists between youth with EDs, Vitamin D deficiency and susceptibility and severity to COVID-19.

An emerging body of research suggests COVID-19 outcomes are worse amongst those suffering from obesity with a significant number of obese and overweight patients requiring intensive care [28]. As obesity is frequency seen amongst those with BED and can sometimes be associated with BN, these patients could be considered higher risk of severe COVID-19 infection.

The medical co-morbidities associated with EDs make this group of patients more vulnerable to COVID-19 which places additional stress on carers as they attempt to shield their child to minimize unnecessary potential exposure to the virus.

### Exacerbation of psychiatric co-morbidity amongst youth with eating disorders

Psychiatric co-morbidity, including depression, anxiety, and obsessive–compulsive disorder, frequently co-occur with EDs. Previous studies have found higher rates of suicide and self-harm amongst patients with EDs, with those with AN 31 times more likely to end their life by suicide compared to the general population [29]. Rates of self-harm amongst patients with BN is 3–20% [30].

Initial reports indicate fears of virus contamination have fueled health anxiety amongst patients with an ED and obsessive–compulsive behaviors around food and exercise have increased during the COVID-19 period [8]. Clinicians working within a pediatric tertiary hospital in Singapore reported a worsening of health-related fears and phobias amongst patients with EDs putatively linked to the pandemic [31].

Seeing their child’s mental health further deteriorate because of restrictions and associated social disconnectedness undoubtedly impacts the psychological well-being of the carer, as evidenced by personal reports [8]. A cross-sectional study amongst ED and non-ED carers showed significantly higher levels of depression and anxiety amongst ED carers during the pandemic in China [32]. Understanding and identifying key factors contributing to the increase in psychological distress amongst both patients with EDs and their carers during COVID-19 is essential to provide appropriate supports and to intervene at an early stage.

### Increased practical demands placed on carers

COVID-19 related food availability and insecurity were initial fears that had relevance to those with disordered eating. Individuals with AN or ARFID worried about access to their ‘safe foods,’ placing additional pressure on carers to search for these foods amongst already bare supermarket shelves [7]. Media highlighting food insecurity may have contributed to hoarding of food and subsequent bingeing amongst those with BED or BN. Carers needed to protect their child from the constant presence of food items due to home confinement and cope with the impact of bingeing depleting household food items when shopping to replace them was difficult.

Lack of daily structure and routine alongside additional time spent within a triggering environment has been cited as being particularly stressful for individuals with ED symptoms during COVID-19 [33]. With community programs placed on hold, youth with EDs are spending more time at home in unstructured settings. As a result, ED cognitions and behaviors may be strengthened and increased carer vigilance for compensatory behaviors such as excessive exercise or self-induced vomiting is required.

Social media usage has soared over the course of the pandemic with socializing online becoming the norm. Of concern, patients with an ED have experienced the social media attention and discussion around risks of weight-gain during lockdown to be particularly triggering [8]. They are also at risk of increased exposure to cyber-bullying, a known risk for the development of psychopathology, including suicidality [34]. While carers often struggle at the best of times to keep their child occupied and distracted from their ED cognitions and behaviors, the pandemic has created unprecedented challenges whereby carers in the absence of usual distractions are having to spend more of their time at home monitoring and supporting their child. The additional carer burden is evident.

### Social isolation, heightened expressed emotion and decreased social support

Prolonged isolation can profoundly affect mood and is a potential risk factor for premature morbidity and mortality [35]. The terms isolation and quarantine have been used interchangeably during the pandemic. A recent review published in the Lancet [36] examined the psychological impact of quarantine on individuals during previous pandemics such as SARS, Ebola and H1N1. They concluded the psychological impact of quarantine was wide-ranging, substantial, and potentially long-lasting. Most studies reported negative psychological effects including post-traumatic stress symptoms, confusion and anger. Common stressors included longer quarantine duration, infection fears, frustration, financial loss and boredom. With quarantine and stay at home orders being used to contain the COVID-19 virus, carers are required to spend more time than usual at home, in some cases working and supervising home schooling, removed from other more pleasurable and mood elevating experiences. These requirements have contributed to stress amongst parents and are likely to be amplified when a parent is also required to assume an increasing responsible role as carer.

Living with and caring for a child with an ED can facilitate a high level of conflict and dysfunction within the family environment [37]. Expressed emotion (EE) has been defined as attitudes and behaviors communicated towards the child with an ED and includes critical comments, hostility and emotional over-involvement [18, 38]. ED research has highlighted the adverse impact of heighted EE on the patient, negatively influencing ED treatment adherence and recovery and exacerbating carer burden [18, 39–41]. Given the intensity of family confinement during the COVID-19 lockdown, the likelihood of raised EE is high.

Prior to COVID-19, carers may have relied on friends or external family members as a source of psychosocial support. Loss of employment, or a decision to work from home given their child’s physical vulnerability may further reduce the amount of contact carers have with others outside the home. Furthermore, the confinement of all family members to the home and the need to balance work and family life places increased pressures on all. For family members of a young person with an ED, mealtimes may become particularly stressful and lead to emotional outbursts and high EE. Given the link between high EE and poor outcomes amongst patients with an ED, it is not surprising that emerging research suggests patients with an ED are experiencing a deterioration or recurrence of ED symptoms during COVID-19 [42]. Carers may feel imprisoned and isolated within their home trying to balance the needs of their family, while their usual outlets to manage stress and psychosocial support have been curtailed due to the pandemic.

## Supporting carers of youth with an eating disorder during and after the COVID-19 pandemic

Failure to identify, acknowledge and address carer burden runs the risk of further decreasing the quality of life of carers and subsequently reducing the efficacy of care they are able to access for and provide to their child [13]. Research shows well-supported carers are more likely to provide better care, experience enhanced benefits of caregiving and generate savings within the healthcare system [13]. Supporting carers and families of patients with EDs during and after the COVID-19 pandemic is vital to optimize the prognosis of ED recovery and wellbeing of families.

In many countries, families are now a pivotal part of ED treatment. This shift means families are now increasingly involved in the provision of their child’s care and are an integral part of recovery. Despite Family-based treatment (FBT) being considered gold standard treatment for youth with EDs, many services have recognized the need to augment this with parent support groups as part of each child’s comprehensive care plan. In response to a greater need for more structured, educational and emotional support for carers, BodyWhys, a national voluntary ED organization supporting individuals and their carers in Ireland, developed the Peer-Led Resilience (PiLAR) program in 2014. A mixed methods evaluation provided evidence of efficacy with carers reporting an enhancement in knowledge, skills and emotional well-being [43]. Clinicians also perceived carers as more knowledgeable and confident assuming parental responsibility for treatment progress [43]. Other group interventions for carers of ED patients focusing on psychoeducation [44] self-help [45], and skills training [46], have also demonstrated efficacy in reducing carers’ distress, enhancing self-efficacy and decreasing expressed emotion [32]. Continuing to support carers while minimizing the risk of COVID-19 spread is imperative. This means the modification of existing supportive interventions from face-to-face to online via the use of smartphones and computers needs to be considered.

During the COVID-19 pandemic, there has been an extraordinary growth in the use of telepsychiatry, the delivery of psychological and medical treatment via technological means. Within the ED treatment settings, telepsychiatry has allowed for continued patient monitoring and assessment, prescription of medication and further therapeutic work. Given the combination of heightened medical risk, exacerbation of psychiatric co-morbidity and reduced access to face-to-face ED services during the pandemic, optimization of telepsychiatry is warranted to support the ED patient and their carer. Contrary to expectation, one study showed during the initial peak of COVID-19, some carers of patient with EDs perceived improved ED service provision and a higher standard of support offered through the use of phone calls or video conferences compared to traditional face-to-face ED services [8]. Given the ongoing and indeterminable duration of the pandemic further research will need to be undertaken across various countries to assess the overall impact of telepsychiatry on service provision for patients with EDs and their families. It may be that a blended approach to service provision is optimum, having adequate face-to-face opportunity especially at initial assessment to help determine physical status, and a low tolerance to face-to-face reviews in the presence of weight loss or carer concern. Providing additional sessions online, at carer and young person request, might reduce time constraints attached to in person attendances.

Extending telepsychiatry to support carers of youth with EDs may include online support groups and psycho-educational events. Due to their adaptive, flexible and low-cost nature, the implementation of online interventions could be practical and effective form of support offered to carers during the COVID-19 pandemic. Evidence suggests online supportive interventions for carers has been helpful. A randomized controlled trial (RCT) showed superiority of a structured web-based support intervention with limited clinician guidance over ad-hoc usual support from the UK patient and carer organization, BEAT, in reducing carer distress and burden of caregiving [47]. The main finding of this pilot RCT was that the web-based multi-media intervention treatment had a significantly greater positive impact on carers’ levels of anxiety and depression compared to the control intervention. Feedback from those who participated was of an acceptable and useful intervention. Online interventions have the advantage of being accessible at any time without having to make appointments or spending time travelling which may be particularly attractive for carers during COVID-19. Given the unique aspects and restrictions placed on families during COVID-19, additional targeted components such as how to access urgent care and monitor their child’s physical and mental health status from home, already part of clinical services, could be added [22].

Previous studies have identified heightened needs of carers supporting youth with EDs. Previously reported un-met needs have included ‘information about EDs,’ ‘Support from other people/organizations’ and ‘Information received from general practitioners’ [48]. These needs may be exacerbated by limitations placed on services and families during COVID-19 but are also amenable to online and virtual delivery. What is more difficult is to carefully balance risk and benefits associated with face-to-face contact for the patient and carer. A better understanding is needed of the nuances of lockdown and restrictions, and in a bespoke way address the challenges unique to each child and their family. Accepting the real risk of infection from COVID-19, bolstering each child’s immune system and nutritional state becomes even more important than pre-pandemic. Looking for new supports for patients and carers, such as those that can be delivered via telehealth, and considering what or which of these types of interventions might be worth retaining post pandemic is also of particular importance.

## Conclusion

The COVID-19 pandemic poses a threat to the mental health of patients and carers and has placed undue strains on health care providers and service delivery. The adverse impact on those caring for youth with a mental illness and EDs in particular seems to be increased.

Further research is warranted disentangling the impact of COVID-19 in terms of associated restrictions on the youth and family, fear of illness in family members and other non-eating disorder stresses on the family unit. Perspective on increased family presence at home is likely to be different between parents, siblings and patients, and influenced by other social and economic factors. The lived experience of youth and carers during this time of unprecedented challenge is worthy of qualitative exploration. Longitudinal studies examining the incidence and prevalence of youth with EDs following the pandemic is essential given initial reports of disproportionate increased presentations. Client satisfaction studies will allow the experience of altered service provision to be carefully reviewed, from the perspective of both carer and young person, so that these lead to improved delivery and flexibility going forward, without any loss of treatment efficacy.

Given the salient role families play caring for youth with EDs, the voice of the carers needs to be heard as clearly as the voice of the young person. Addressing carer burden during COVID-19 and beyond has the potential to alleviate the suffering of the carer, subsequently contributing to more positive treatment outcomes amongst youth with EDs.

## Data Availability

Not applicable.

## Abbreviations

ED: Eating Disorder
AN: Anorexia Nervosa
BED: Binge Eating Disorder
BN: Bulimia Nervosa
ARFID: Avoidant Restrictive Food Intake Disorder
SARS: Southeast Asian Respiratory Syndrome
H1N1: H1N1 Influenza
PiLAR: Peer-Led-Resilience Program
FBT: Family Based Therapy
RCT: Randomized Controlled Trial
